# Visible-light-stimulated synaptic InGaZnO phototransistors enabled by wavelength-tunable perovskite quantum dots†

**DOI:** 10.1039/d1na00410g

**Published:** 2021-08-03

**Authors:** Zhilong Xin, Yang Tan, Tong Chen, Emad Iranmanesh, Lei Li, Kuan-Chang Chang, Shengdong Zhang, Chuan Liu, Hang Zhou

**Affiliations:** School of Electronic and Computer Engineering, Peking University Shenzhen Graduate School Shenzhen 518055 China kcchang@pkusz.edu.cn zhouh81@pkusz.edu.cn; State Key Laboratory of Optoelectronic Materials and Technologies, School of Electronics and Information Technology, Sun Yat-Sen University Guangzhou 510006 China

## Abstract

Neuromorphic vision sensors are designed to mimic the human visual system, which allows image recognition with low power computational requirements. Photonic synaptic devices are one of the most viable building blocks for constructing neuromorphic vision sensors. Herein, a photonic synaptic sensor based on an inorganic perovskite quantum dot (QD) embedded InGaZnO (IGZO) thin-film phototransistor is demonstrated. The photodetection wavelength ranges of the transistor can be adjusted by changing the halogen ions (Cl, Br) of the perovskite QDs. Under low intensity 450 and 550 nm illumination, the CsPbBr_3_ QD embedded phototransistor sensor shows a responsivity of 6.7 × 10^2^ and 4.2 × 10^−2^ A W^−1^, respectively. The perovskite QD embedded transistor not only presents high responsivity to visible light, but also features excellent synaptic behavior, including an excitatory postsynaptic current (EPSC), pair-pulse facilitation (PPF), long-term memory, and memory erasure through gate voltage regulation. Moreover, the sensor fabrication process in this work is compatible with conventional photolithography processes. Taking these merits into account, the proposed QD embedded IGZO transistor presents a promising route by which to construct artificial visual sensors with color-distinguishable optical signal sensing and processing.

## Abstract 2

The detected wavelength of perovskite quantum dots embedded in IGZO TFT can be tuned by replacing the quantum dot halogen ions. It is expected that a color-distinguishable artificial human vision system can be developed.

## Graphical abstract

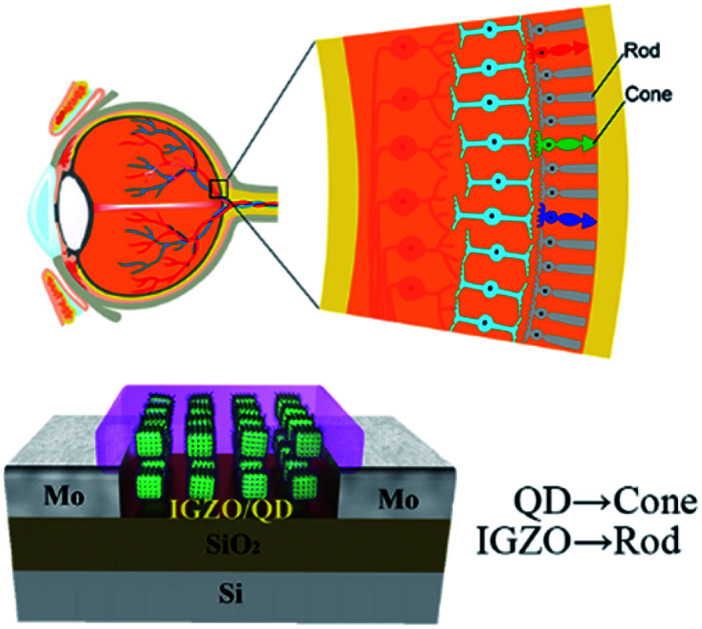

## Introduction

**Fig. 1:**
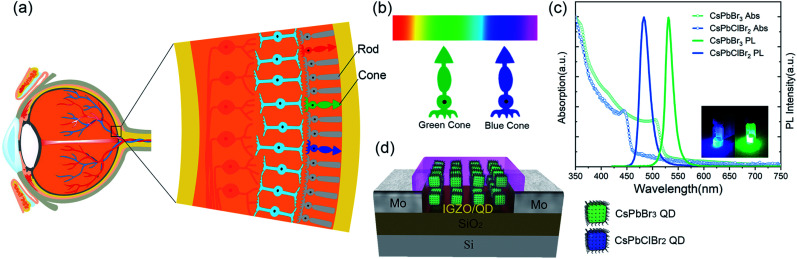
(a) The structure of the human eye retina. The color perception refers to the cones. (b) The absorption peaks of the blue and green cone cells in the human eye. (c) Absorption and photoluminescence (PL) spectra of CsPbClBr_2_ QDs and CsPbBr_3_ QDs. Inset: QD solutions under UV light. (d) Schematic diagram of a perovskite QD/IGZO phototransistor.

Although silicon-based digital image sensors are widely used nowadays, our modern imaging sensor systems are still far from being convergent with the human eye visual system. The photosensitive and acquisition units are basically separated in a typical camera. These conventional digital machine vision systems tend to have excessive power consumption and require large memory space. Neuromorphic vision sensors are perhaps one of the most attractive future vision technologies.^1–4^ Neuromorphic vision sensors are meant to share receiving and computing nodes along with storage elements in physical space, and can process information in real time and in parallel. Such sensors are in wide demand due to the reduction of the computational load required for visual perception by extracting only the relevant information in the post-processing stages.^5^ Taking a glance of the human visual system illustrates that the human retina consists of rods and three types of cones: red, green and blue, as shown in Fig. 1a. The cones and rods are sensitive to color and light intensity, respectively. Color perception is determined by the human brain, wherein various cone cells function as color signal input. The three types of color sensitive cone cells (red, green and blue, as shown in Fig. 1b) are responsive to incident light with wavelengths centered at 570, 540, and 440 nm, respectively. It is interesting to note that the cone cells are actually sandwiched in ‘color-blind’ rod cells. The response and recovery times of photoreceptors in the human retina ranges from 40 to 150 ms.^6^ The presence of high light intensity prolongs the persistence of vision generally for more than 0.1 s. Inspired by the human vision system, combined with advanced artificial intelligence technology, an artificial vision system with perception, processing and memory has captured the wide attention of researchers.^7–9^ Synaptic devices for color discrimination are expected to simulate human vision systems to achieve color-sensitive neural network systems. Here, we imitate the photoreceptor cell structure in the human retina by constructing a photonic synaptic sensor based on a halogen-adjustable perovskite quantum dot embedded InGaZnO (IGZO) thin-film transistor. The perovskite QDs of the proposed sensor imitate color recognition cone cells, which are sandwiched in the low leakage current IGZO layers, enabling low power consumption and high sensitivity towards visible light.

The high field-effect carrier mobility, high transparency, and capability of fabrication on flexible substrates makes IGZO a candidate to be utilized in a wide range of applications, from display panels to logic circuits.^10,11^ The mobility of the metal oxide semiconductor, IGZO, is higher than that of amorphous silicon, which meets the requirements where large driving current density is needed, such as organic light-emitting diode (OLED) displays.^12,13^ Nonetheless, with a wide bandgap of 3.5 eV, IGZO is only sensitive to the ultraviolet wavelength region, limiting its application as a visible light sensor.^14^ To extend its photodetection range, hybrid IGZO phototransistors with various light absorption layers like metal oxide semiconductors, polymer semiconductors and quantum dots have been reported.^15–18^ Inorganic lead halide perovskite (CsPbX_3_, X = Cl, Br, I) quantum dots (QDs), which possess higher stability than their thin-film counterparts, have gained extensive research interest in the field of light-emitting diodes and photodetectors.^19–24^ Previously, we have shown enhancement in photoresponsivity by capping IGZO with perovskite QDs.^18^ Perovskite QDs seem attractive enough in this case due to their tunable-wavelength capability. Previous reports have also recently addressed halide perovskite based photonic synapsis, which interestingly emulate both synaptic plasticity and learning processes.^25–28^

Our device configuration of the perovskite QD embedded IGZO phototransistor features stacks of IGZO/QDs/IGZO in the channel region. This sandwich structure allows QDs to be protected from oxygen, water and organic solvent attack. The stacked layers can then be patterned into small devices *via* conventional photolithography. The patterning capability is essential for designing a high-resolution imaging pixel array. Short-term plasticity (STP) behavior such as excitatory postsynaptic current (EPSC) and paired-pulse facilitation (PPF) are demonstrated. Moreover, the transformation from short-term to long-term plasticity (LTP) can be realized by adjusting the shape of the light pulse. Tuning the gate voltage pulse results in an inhibitory postsynaptic current (IPSC), which thereafter erases the memory-based current.

## Results and discussion

### IGZO transistor embedded with perovskite quantum dots

As shown in Fig. 1c, the emission peaks of the CsPbClBr_2_ and CsPbBr_3_ QDs are centered at 483 and 531 nm, with full width at half maxima (FWHM) of 22 and 17 nm, respectively. The FWHM of the CsPbClBr_2_ QDs is slightly larger, which is expected due to the larger absorption tail of its sub-band gap. The X-ray diffraction (XRD) pattern of the perovskite QDs are in accordance with the CsPbBr_3_ cubic lattices (PDF# 54-0752), as shown in Fig. S1.† The right shift in the XRD diffraction peak of the CsPbClBr_2_ QDs can be attributed to a decrease in the lattice constant due to the doping of chloride ions.^29^ The transmission electron microscopy (TEM) analysis in Fig. S2† shows that the CsPbBr_3_ QDs samples are in cuboid shapes with an average size of ∼10 nm. The lattice fringes of the CsPbBr_3_ QDs display an interplanar spacing of 0.29 nm, which matches well with the (100) planes.

**Fig. 2:**
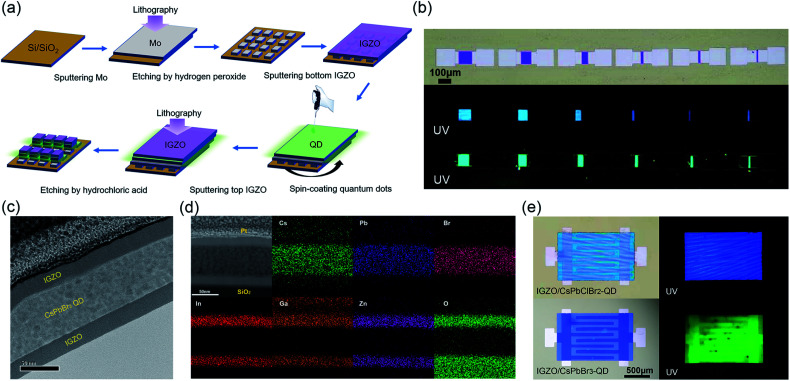
(a) The fabrication process of IGZO/QD TFT arrays *via* photolithography patterning. (b) Optical microscopy images of IGZO/CsPbClBr_2_-QD and IGZO/CsPbBr_3_-QD phototransistor arrays under UV light. From left to right: devices with channel lengths varying from 100 to 12 μm. (c) TEM images of a cross-sectional view of a IGZO/CsPbBr_3_-QD phototransistor in the channel region. (d) EDS mapping images of the Cs, Pb, Br, In, Ga, Zn and O elements of the channel region. (e) Top view of the phototransistor for synaptic response testing with interdigital electrodes.

A schematic diagram of the device is shown in Fig. 1d and the fabrication process is schematically shown in Fig. 2a. Perovskite quantum dots (CsPbX_3_, X = Cl, Br) were synthesized experimentally *via* an improved process at room temperature in air, and were spin-coated on the bottom IGZO layer. Then, the QDs were capped by another upper IGZO layer. A positive photoresist (PR1-2000A1) was spin-coated onto the upper IGZO layer, followed by a standard photolithography process to define the active channel region. Details of the fabrication process can be found in the device fabrication section. The residual photoresist was removed by acetone. The different channel lengths of the QD embedded IGZO transistors are shown in Fig. 2b. The smallest channel length was 12 μm in the experiments. Under UV light, the IGZO/QDs/IGZO region exhibits strong blue or green fluorescence light, as shown in Fig. 2b. The emission peaks of the channel region are consistent with the photoluminescence (PL) spectra of the CsPbClBr_2_ and CsPbBr_3_ QDs shown in Fig. 1c. This indicates that the perovskite QDs have high tolerance to the fabrication process.

A cross-sectional TEM image of the channel region is presented in Fig. 2c. This figure shows that the CsPbBr_3_ nanoparticles are densely and uniformly packed and embedded in the middle of the two layers of IGZO. The thickness of the spin-coated QD film was ∼60 nm. The active region was also analyzed by TEM using a microscope coupled with energy-dispersive X-ray spectroscopy (EDS) to confirm the composition of the thin film, as shown in Fig. 2d.

**Fig. 3:**
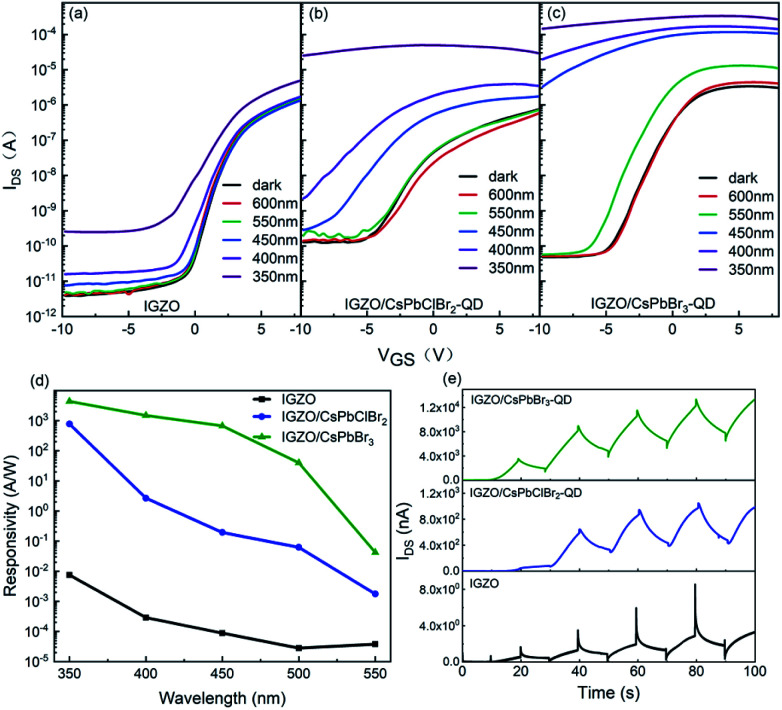
Transfer characteristics of the (a) IGZO, (b) IGZO/CsPbClBr_2_-QD, and (c) IGZO/CsPbBr_3_-QD phototransistors (*V*_D_ = 4 V) at different wavelengths of 3.5μW cm^−2^ light intensity. (d) Responsivity of the IGZO, IGZO/CsPbClBr_2_-QDs, and IGZO/CsPbBr_3_-QDs at *V*_D_ = 4 V when the *V*_GS_ is at the maximum *I*_light_/*I*_dark_ ratio. (e) Transient response of IGZO, IGZO/CsPbClBr_2_-QD, and IGZO/CsPbBr_3_-QD at *V*_D_ = 4 V when the *V*_GS_ is at the maximum *I*_light_/*I*_dark_ ratio (450 nm, 3.5 μW cm^−2^).

In order to achieve a high *I*_on_/*I*_off_ ratio, phototransistors with interdigitated source and drain electrodes were fabricated, as shown in Fig. 2e. The effective channel width and length ratio was 8200/100. Fig. 3 shows the transfer characteristics (*V*_D_ = 4 V) of IGZO (a), the CsPbClBr_2_ QD embedded IGZO TFT (IGZO/CsPbClBr_2_-QD) (b), and the CsPbBr_3_ embedded IGZO TFT (IGZO/CsPbBr_3_-QD) (c) in the dark and under illumination. The light intensities were kept constant at 3.5 μW cm^−2^. The IGZO/CsPbClBr_2_-QD TFT and IGZO/CsPbBr_3_-QD TFT shows significant response towards light below 500 and 550 nm, while IGZO TFT only responds to light below 450 nm. Compared with pristine IGZO TFT, the embedding of QDs shifts the threshold voltage of TFT negatively by ∼4 V, and the off-state currents increase noticeably, which is expected due to charge transfer at the IGZO/QD interface.^30^ Under illumination, electrons are excited to the conduction band of perovskite QDs, and the remaining holes cause a photogating effect.^31^ In the cut-off region, the threshold voltage of IGZO/CsPbBr_3_-QD TFT shifts negatively when exposed to light, resulting in a large light-to-dark current ratio. The off-state dark currents of IGZO/CsPbBr_3_-QD TFT and IGZO/CsPbClBr_2_-QD TFT are 4.8 × 10^−11^ A and 1.3 × 10^−10^ A. The *I*_light_/*I*_dark_ ratio values of the IGZO/CsPbBr_3_-QDs at different *V*_GS_ are shown in Fig. S3a.† The large *I*_light_/*I*_dark_ ratio of the device indicates that it can undergo photoelectric conversion under 550 nm radiation. To evaluate the light response of the hybrid phototransistors, the responsivities of IGZO, IGZO/CsPbClBr_2_-QD, and IGZO/CsPbBr_3_-QD TFT were then calculated. The *V*_D_ was biased at 4 V, and the *V*_GS_ was biased at the maximum *I*_light_/*I*_dark_ ratio. As shown in Fig. 3d, the IGZO/CsPbBr_3_-QD TFT responsivity reaches 4.4 × 10^3^ A W^−1^ in the ultraviolet wavelength region. A performance comparison of the IGZO/CsPbClBr_2_-QD and IGZO/CsPbBr_3_-QD TFTs are listed in Table 1. According to the analysis in Table 1 and Fig. 3b and c, the IGZO/CsPbClBr_2_-QD TFT shows little response to 550 nm light. The dependence of the device on different wavelengths is due to the absorption of different light wavelengths by the QDs. With an increase in the wavelength, the responsivity decreases gradually. The optical detection performance of the embedded CsPbClBr_2_ QDs is slightly weaker than that of the CsPbBr_3_ QD device, as the CsPbBr_3_ QDs have a narrower bandgap and larger absorption coefficient. On the other hand, the green CsPbBr_3_ QDs are more stable and exhibit a higher photoluminescence quantum yield.^32^Table 1 lists the color recognition of the three IGZO devices. In other words, control over the types of QDs in the sandwiched channel forms a long-lasting color-sensitive photoresponse.

**Table 1:** Photoresponse characteristics comparison between the two TFTs and IGZO

	Wavelength [nm]	*V*_GS_ (V)	*I*_light_/*I*_dark_	Responsivity [A W^−1^]
IGZO	450	−2.9	1.68	8.78 × 10^−5^
	550		1.29	3.81 × 10^−5^
IGZO/CsPbClBr_2_-QD	450	−4.4	6.32 × 10	1.94 × 10^−1^
	550		1.57	1.761 × 10^−3^
IGZO/CsPbBr_3_-QD	450	−5	2.51 × 10^5^	6.70 × 10^2^
	550		1.69 × 10	4.2 × 10^−2^

Fig. S3b† reveals that there is a steady rise in the photocurrent as the light intensity increases under illumination with 450 nm light. The current is not saturated and maintained good linearity when tests were carried out under weak light intensity from 10–25 μW cm^−2^. This is strong proof that the device has good ability to detect low light intensity. The transient responses of the three devices are shown in Fig. 3e. The IGZO TFT photocurrent slowly rises under 450 nm illumination and slowly decreases in the dark for persistent photoconductivity (PPC) caused by oxygen vacancies in IGZO.^33,34^ As shown in Fig. S4,† the off-state current of the IGZO/CsPbBr_3_-QDs decreases by an order of magnitude after 45 days. This is probably related to the repair of oxygen vacancies in IGZO by air. At the same time, it still has a high response under 450 nm light. The CsPbClBr_2_ and CsPbBr_3_ QDs devices were tested ∼8 months later, as shown in Fig. S5.† Both of them showed an obvious photoresponse, demonstrating the stability of the devices.

### Biosynaptic characteristics of the IGZO/CsPbBr_3_-QDs phototransistors

**Fig. 4:**
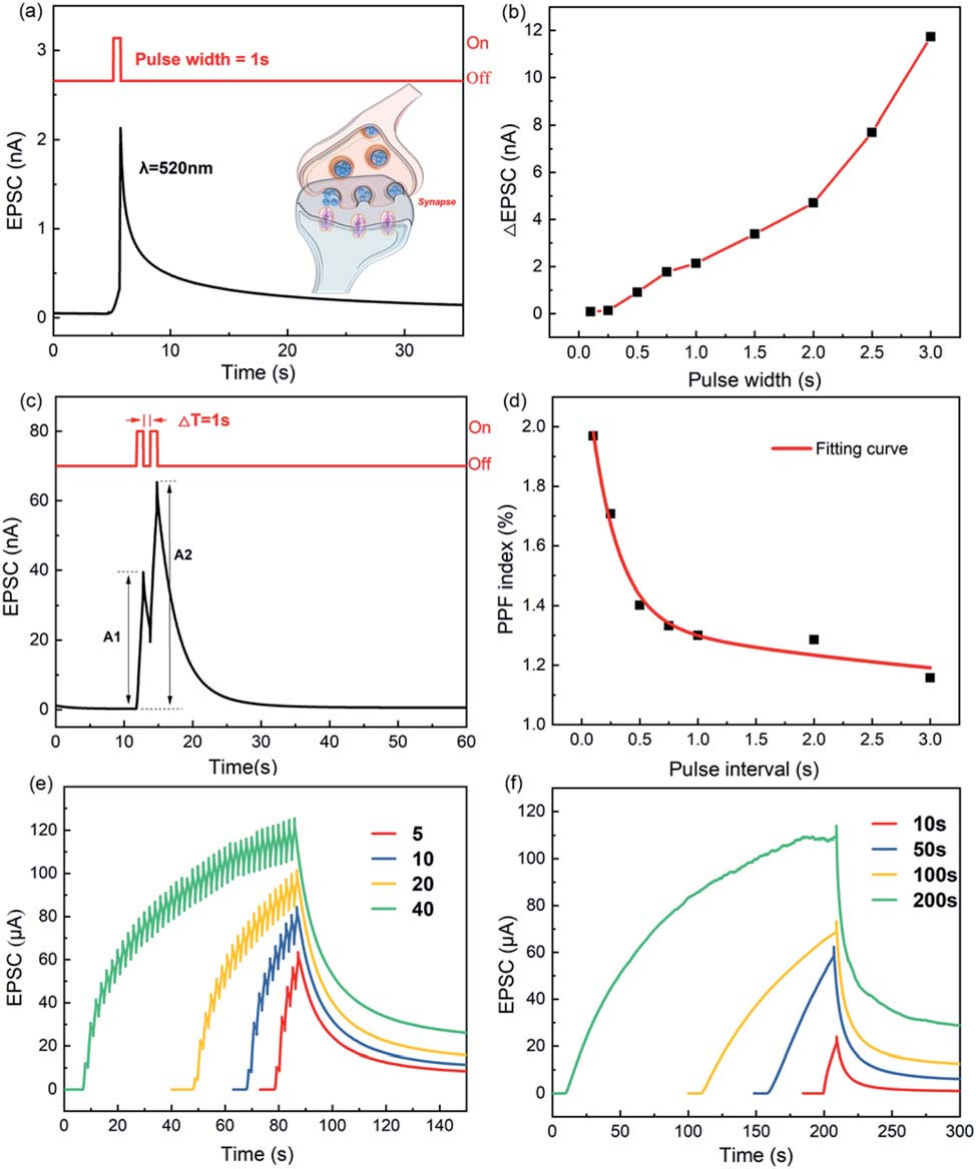
(a) The EPSC triggered by optical pulse (520 nm, 1 s) at a drain voltage of 10 mV and a gate voltage of −20 V. Inset: Schematic diagram of the signal propagation of neurons. (b) The variation in EPSC as a function of the optical pulse width. (c) EPSC induced by two 1 s light spikes (520 nm, Δ*T* = 1 s), and (d) PPF index at different pulse intervals. (e) The change from short-term plasticity to long-term plasticity by changing the number of light (450 nm) pulses in the range of 5–40 pulses, and (f) by changing the light illumination time from 10 to 200 s (*V*_D_ = 4 V and *V*_GS_ = −15 V).

Synapses in biology can transmit information from the presynaptic neuron to the postsynaptic neuron. The signal transmission between neurons is first received by the dendrites of the preneurons. Thereafter, the cell bodies integrate and encode information. The pulse signals are then sent through axons. 520 nm visible light is regarded as the input signal of presynaptic neurons, and the potential change of the postsynaptic neurons is represented by the drain current, *I*_DS_. Fig. 4a shows that the EPSC of the IGZO/CsPbBr_3_ QDs device is excited by a 520 nm optical pulse lasting for 1 s and then slowly returns to the initial value with gate and drain voltages of −20 V and 10 mV. The power consumption of one event in this test is 1.35 pW. The delay in the rise/fall time in the light and dark is similar to the postsynaptic potential caused by the difference in the opening times of the Na^+^ and K^+^ channels.^35^ The ΔEPSC increases with an increase in the light pulse time, as shown in Fig. 4b. It is indispensable to note that there is a slight deviation in the curve and amplitude under different light intensities. Paired pulse facilitation (PPF) refers to the phenomenon of enhanced response after two consecutive stimuli, which can be expressed using the following equation:1
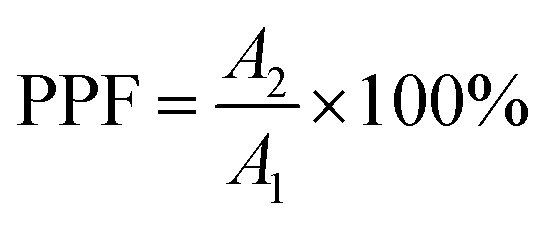
where *A*_1_ and *A*_2_ are the currents generated by the first and second optical pulses, respectively, as shown in Fig. 4c. With an increase in the pulse interval, the PPF index decreases gradually, which can be fitted by a double exponential function of biological synapses:2
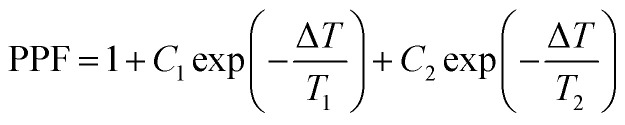
where *C*_1_ and *C*_2_ represent the original facilitation magnitude of the slow and rapid phases, and *T*_1_ and *T*_2_ represent the characteristic relaxation times of the slow and rapid phases.^36^ In our example, the PPF with an interval of 0.1–3 s can be fitted to the red line in Fig. 4d. The parameters *C*_1_, *C*_2_, *T*_1_, and *T*_2_ fitted using the equation are 97%, 35%, 238 ms, and 5 s, respectively. Fig. 4e and f further demonstrate the learning and memorizing capability of this photonic synaptic device under stimulated light (450 nm) according to various pulse numbers and pulse times. The increase in both the number of pulses or pulse duration results in EPSC enhancement and maintains a certain conductivity or synaptic weight over a long time period. This is simply referred to as learning and memory in biology.

It is well-known that the pristine IGZO TFT inherits persistent photoconductance (PPC) behavior under UV exposure. The localized ionized oxygen vacancies (V_O_^2+^) of IGZO inhibit the recovery of dark resistivity after UV exposure,^37^ and the ionized oxygen vacancies can be hardly generated under visible light. The visible-light response is apparently due to the embedded QDs. However, the recombination of electrons and holes is very fast in the QDs, as the PL of the QDs consequently attains a very short decay time range in the nanoseconds.^26^ Hence, the origin of the synaptic behavior under visible light pulsing requires further investigation. Fig. S6† shows that there is no significant difference in the O 1s XPS spectra of the IGZO and IGZO/CsPbBr_3_-QD films before and after visible light irradiation. There is a huge difference in the *I*_DS_ values of the IGZO and IGZO/QD devices under visible light, indicating that the ionized oxygen vacancies are not primarily responsible for the current contribution under visible light. We suppose that certain amounts of photogenerated electrons in the QDs overcome the barriers of the ligand and transfer to the IGZO layer, as shown in Fig. S7.† Due to the large surface-to-volume ratio of low-dimensional perovskite QDs, carriers are trapped by defects near to the ligands, which slow down the recombination rate of photogenerated carries and produce an additional electric field to modulate the channel conductance.^29^ Therefore, the trapped charge also results in PPC just like the ionized oxygen vacancies in the IGZO layer.

**Fig. 5:**
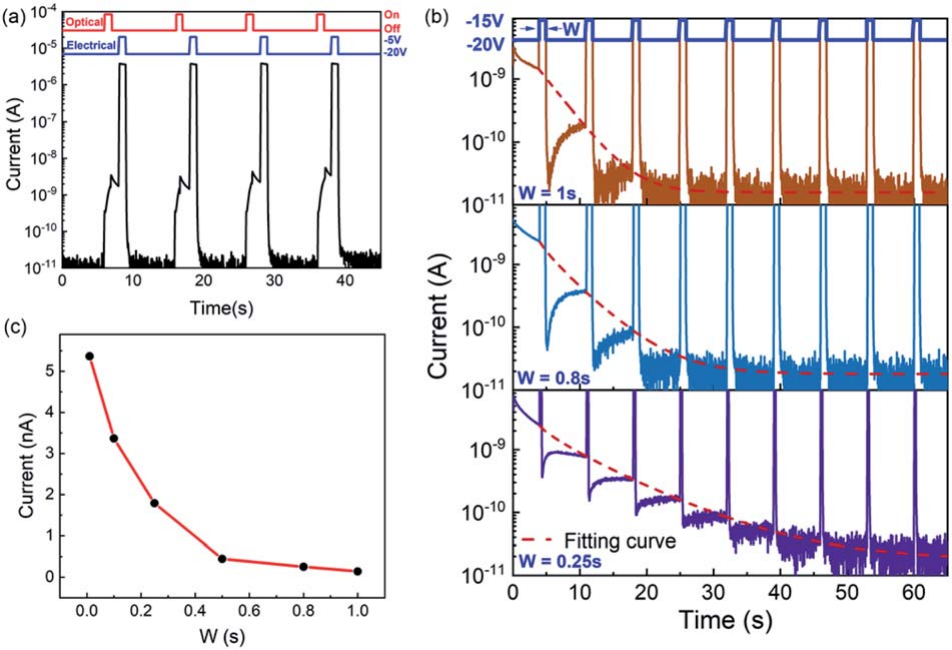
(a) Erasing of the electrical pulse (*V*_GS_ = −20 V → −5 V, 1 s) after optical pulsing (500 nm, 1 s) with a drain voltage of 10 mV and a gate voltage of −20 V. (b) Inhibition of different electric pulse widths (*V*_GS_ = −20 V → −15 V, *W* = 1 s, 0.8 s, and 0.25 s). (c) The current at 10 s under the test conditions of figure (b). Inhibition of different gate pulse widths (0.1–1 s, *V*_GS_ = −20 to −15 V) at a drain voltage of 10 mV and a gate voltage of −20 V.

The forward gate voltage pulse is used to control the activity of postsynaptic currents. As shown in Fig. 5a, a sudden gate electrical pulse from −20 to −5 V is applied after optical pulsing to reset the current. If the amplitude of the electrical pulse is modulated, the inhibitory postsynaptic potential of biological synapses can also be achieved. As shown in Fig. 5b, the applied gate pulses from −20 to −15 V can suppress the postsynaptic current, and we can also adjust the pulse width *W* to control the trend in the postsynaptic current.

The change in the current regulated by electric pulsing is regarded as the inhibition of the EPSC, which can be fitted using the stretched exponential function:3
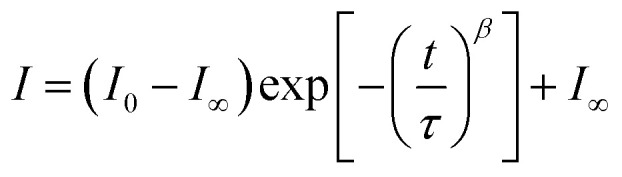
where *I*_0_, *I*_∞_ are the initial and final postsynaptic currents at steady state, and *β* is a stretch index between 0 and 1. *τ*, the retention time, can be characterized as the forgetting rate. Electrical pulses with pulse widths of 1, 0.8, and 0.25 s are applied every 7 s, and the retention times, *τ*, are 3.22, 3.98, and 5.96 s, respectively. The effect of the single pulse width (*W*) on the postsynaptic current is shown in Fig. 5c.

The postsynaptic current can be erased or suppressed by modulating the size and width of the gate pulse. When the gate pulse is positive, the Fermi level in the active region is elevated, and the electrons in the conduction band tend to recombine at a faster rate, whether excited by the localized oxygen vacancy of IGZO under ultraviolet light or isolated by the ligand barrier of the perovskite QDs under visible light. Therefore, the utilization of a positive gate pulse allows extra control of the persistence of the light simulated signal.

## Conclusions

An IGZO phototransistor with embedded perovskite QDs was proved to be an effective UV-visible detector. Hybrid TFT devices not only enhance the photocurrent, but also extend the response wavelength to visible light. Different visible light detection can be realized by adjusting the bandgap of perovskite QDs. The proposed IGZO/QDs/IGZO sandwich structure ensures the fabrication of a small pixel device *via* conventional photolithography. The perovskite QD-embedded transistor shows excellent synaptic behavior, including EPSC, PPF, long-term memory, and memory erasure through gate voltage regulation. These results testify the novelty of the proposed phototransistor as a visual neural device.

## Experimental section

### Device fabrication

A Si wafer covered with 200 nm SiO_2_ was successively ultrasonicated in acetone, alcohol, and deionized water. To fabricate coplanar IGZO TFT, an 80 nm-thick molybdenum inter-digital source and drain electrodes were sputtered using a direct current (DC) source on a clean Si wafer, followed by common lithography and hydrogen peroxide etching. 20 nm-thick IGZO was then sputtered and annealed at 200 °C for 1 h under an oxygen atmosphere. The synthesis of QDs was improved based on reported methods.^38^ PbX_2_ (X = Cl, Br) was dissolved in oleic acid, *n*-octylamine, and *n*-propanol solution at 90 °C to generate a mixed homogeneous solution. Then, a lead precursor was injected into cesium acetate in a mixture of *n*-hexane and *n*-propanol. Uniform particles were centrifuged and re-dispersed in *n*-hexane solution. Dispersed CsPbClBr_2_ or CsPbBr_3_ QDs in *n*-hexane were spin-coated on an IGZO layer at a speed of 1000 rpm for 30 s. Finally, another 20 nm-thick IGZO layer was sputtered on the QD layer. In order to define channels, a positive resist (PR1-2000A1) was spun on the top to protect the active layer and the independent devices were etched using dilute hydrochloric acid. In the next step, the photoresist was removed utilizing acetone. The fabrication process is shown in Fig. 1a. For comparison, a pristine IGZO TFT was fabricated by sputtering 40 nm-thick IGZO using the same process but without QDs.

### Sample measurements

The UV-visible absorption spectra of the QD films were measured by UV-Vis spectroscopy (UV-2600, Shimadzu). Photoluminescence spectra were collected using a portable FX2000-EX spectrometer. XRD patterns were recorded employing a D8 Advance (Bruker) diffractometer. Electrical characteristics and the photoresponse of the phototransistors were tested using a semiconductor parameter analyzer (Agilent B1500) housed in the lab under dark and illumination conditions. The monochromatic light used in the experiments was generated by a xenon lamp through a monochromator (Zolix, Omni-l 3009). We used a power meter (Newport, 1936-R) to measure the incident optical power. TEM images were collected using a JEM-3200FS transmission electron microscope equipped with an EDS detector and a Gatan 994 Ultrascan camera. XPS spectra were obtained using an ESCALAB 250Xi spectrometer.

## Conflicts of interest

There are no conflicts to declare.

## Supplementary Material

NA-003-D1NA00410G-s001
