# Supplementary material for: Visible-light-stimulated synaptic InGaZnO phototransistors enabled by wavelength-tunable perovskite quantum dots
Source: Nanoscale Adv. 2021 Aug 3;3(17):5046–52. doi: 10.1039/d1na00410g (PMC9417670; doi:10.1039/d1na00410g)
Supplement: NA-003-D1NA00410G-s001 [file NA-003-D1NA00410G-s001.pdf]

Electronic Supplementary Material (ESI) for Nanoscale Advances.
This journal is © The Royal Society of Chemistry 2021

Supporting Information

Visible-light-stimulated synaptic InGaZnO phototransistors enabled by wavelength-tunable perovskite quantum dots

Zhilong Xin , Yang Tan, Tong Chen, Emad Iranmanesh, Lei Li, Kuan-Chang Chang, Shengdong Zhang, Chuan Liu, Hang Zhou**

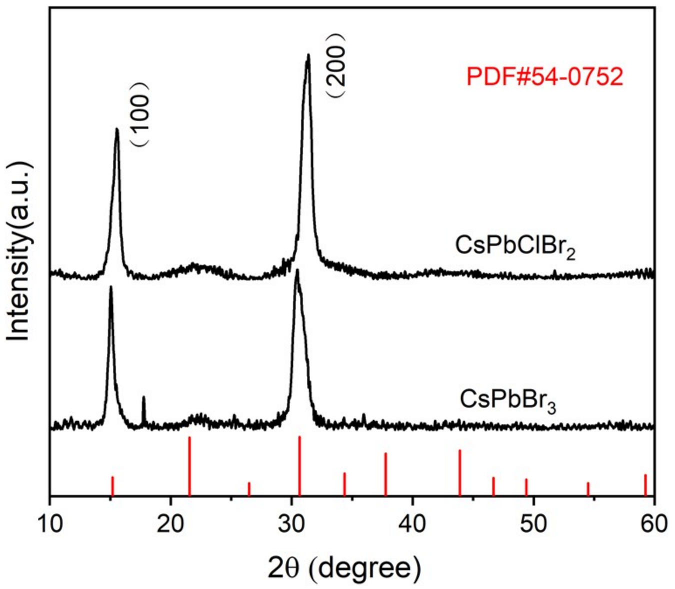

Figure S1. XRD pattern of $CsPbClBr_2$ QDs and $CsPbBr_3$ QDs.

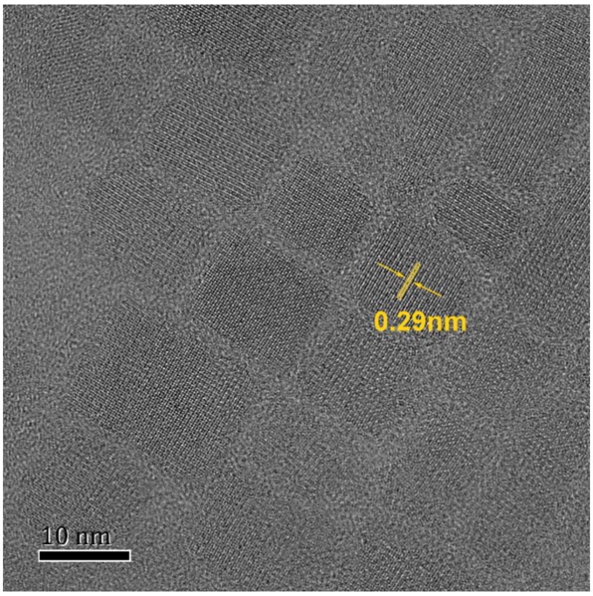

FigureS2. TEM image of $CsPbBr_3$ QD

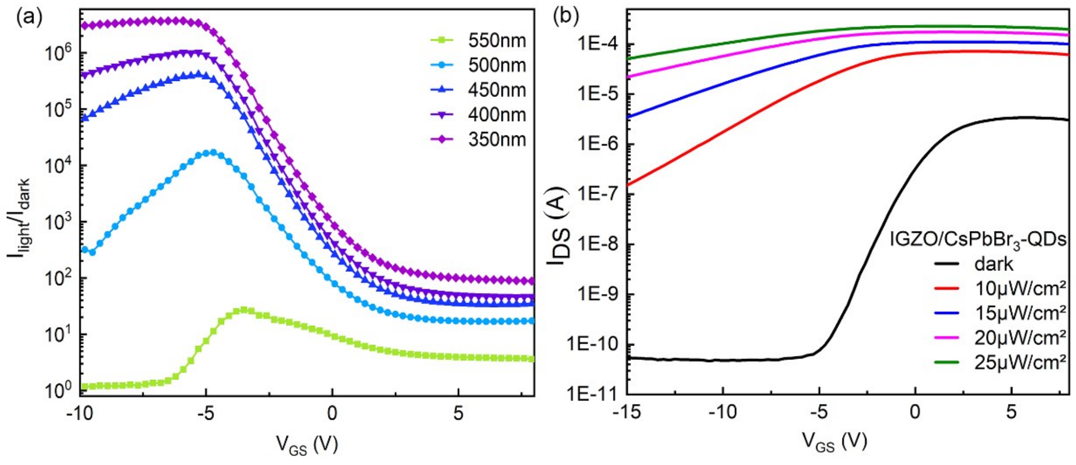

Figure S3 (a) The I_{light}/I_{dark} ratio values of IGZO/$CsPbBr_3$-QDs at different wavelengths of 3.5μw cm^{-2} light intensity (V_D = 4V). (b) Transfer characteristics of IGZO/$CsPbBr_3$-QDs as a function of the light intensity at a fixed illuminating wavelength (450nm).

Table S1 Color recognition by using three IGZO TFTs

	IGZO	IGZO/$CsPbClBr_2$-QD	IGZO/$CsPbBr_3$-QD
UV	On	On	On
Blue	Off	On	On
Green	Off	Off	On

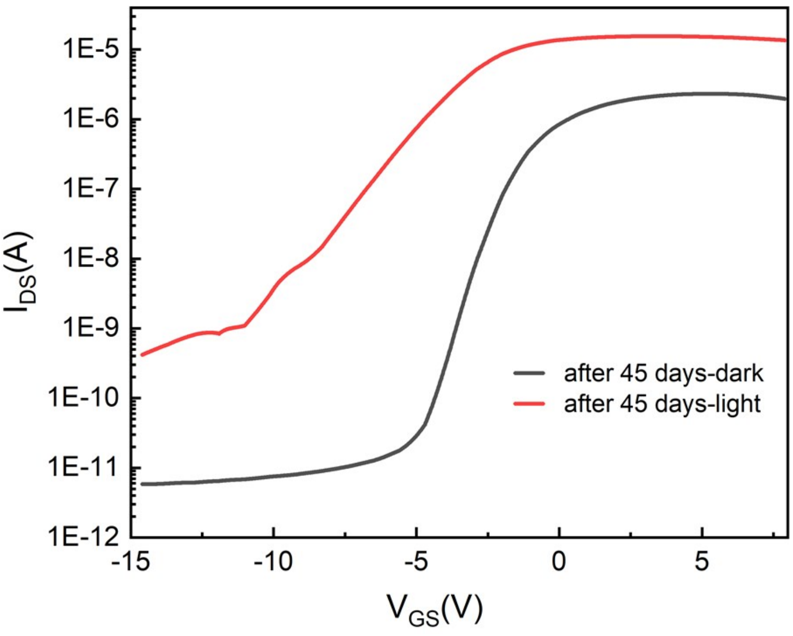

Figure S4. Transfer characteristic of IGZO/$CsPbBr_3$-QDs under dark illumination states (450nm, 3.5μw cm^{-2}) after 45 days.

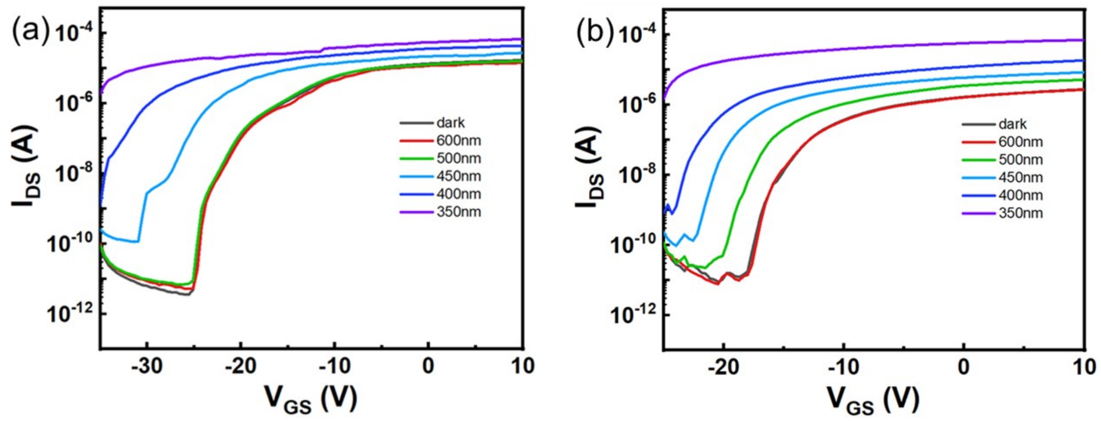

Figure S5. Transfer characteristics of (a) IGZO/CsPbClBr2-QD and (b) IGZO/CsPbBr3-QD phototransistors (VD = 4V) at different wavelengths measured in ~8 months later.

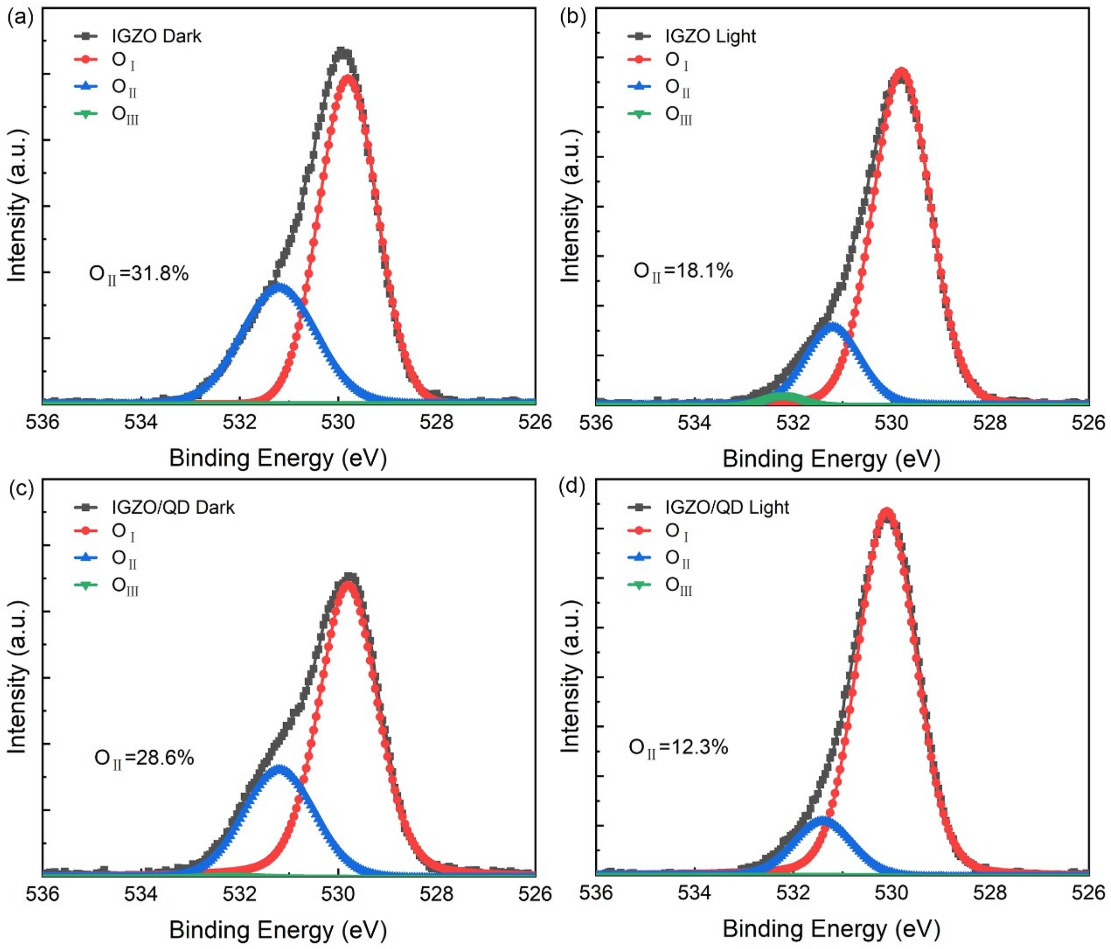

Figure S6. XPS spectra of O 1s of the IGZO and IGZO/$CsPbBr_3$ QDs/IGZO films before and after white light irradiation.

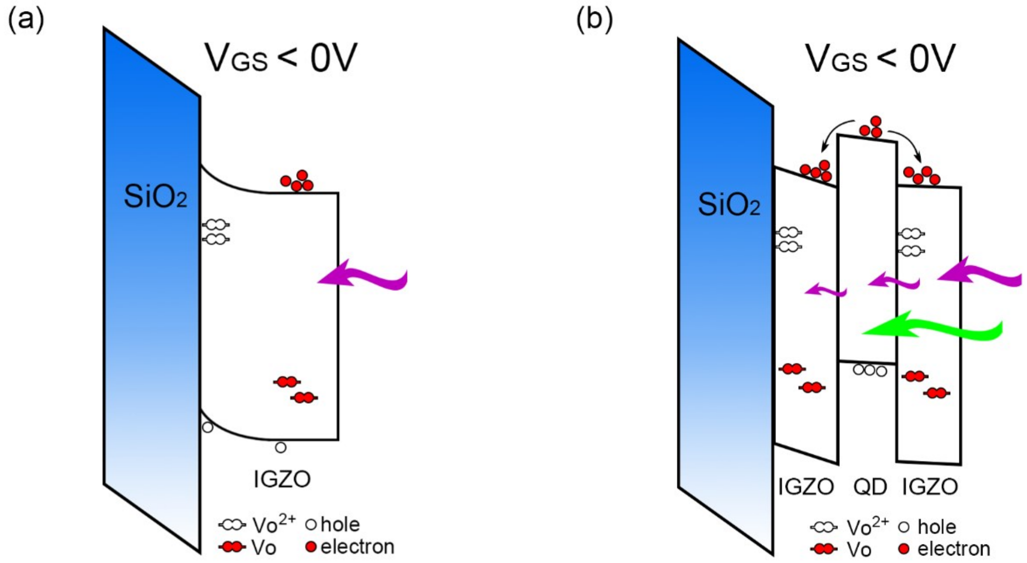

Figure S7. Schematic band diagram of the (a) IGZO device and (b) IGZO/QD device under a negative gate bias and light illumination. The oxygen vacancy Vo^{2+} in the IGZO layer is excited only under UV irradiation. While irradiated by visible light, only the electron and holes in QDs are separated.
